# Evaluation of liquid and powdered forms of polyclonal antibody preparation against *Streptococcus bovis* and *Fusobacterium necrophorum* in cattle adapted or not adapted to highly fermentable carbohydrate diets

**DOI:** 10.5713/ajas.19.0761

**Published:** 2020-04-12

**Authors:** Eduardo Cuellar Orlandi Cassiano, Flavio Perna Junior, Tarley Araújo Barros, Carolina Tobias Marino, Rodrigo Dias Lauritano Pacheco, Fernanda Altieri Ferreira, Danilo Domingues Millen, Mauricio Furlan Martins, Silvana Marina Piccoli Pugine, Mariza Pires de Melo, Karen Ann Beauchemin, Paula Marques Meyer, Mario de Beni Arrigoni, Paulo Henrique Mazza Rodrigues

**Affiliations:** 1 Department of Animal Nutrition and Production, University of São Paulo (FMVZ-USP), Pirassununga, São Paulo 13635-900, Brazil; 2 Embrapa Beef Cattle, Campo Grande, Mato Grosso do Sul 79106-550, Brazil; 3 Mato Grosso State Agricultural and Extension Service, Varzea Grande, Mato Grosso 78115-100, Brazil; 4 Department of Animal Science, São Paulo State University (UNESP), Dracena, São Paulo 17900-000, Brazil; 5 Department of Basic Sciences (ZAB), Faculty of Animal Science and Food Engineering (FZEA-USP), Pirassununga, São Paulo 13635-900, Brazil; 6 Agriculture and Agri-Food Canada, Lethbridge Research and Development Centre, Lethbridge, AB T1J 4B1, Canada; 7 Brazilian Institute of Geography and Statistics (IBGE), Pirassununga, São Paulo 13630-095, Brazil; 8 Department of Animal Breeding and Nutrition, São Paulo State University (FMVZ-UNESP), Botucatu, São Paulo 18618-000, Brazil

**Keywords:** Acute Phase Protein, Feed Additive, Passive Immunization, Real-time Polymerase Chain Reaction, Rumen Fermentation

## Abstract

**Objective:**

Feed additives that modify rumen fermentation can be used to prevent metabolic disturbances such as acidosis and optimize beef cattle production. The study evaluated the effects of liquid and powdered forms of polyclonal antibody preparation (PAP) against *Streptococcus bovis* and *Fusobacterium necrophorum* on rumen fermentation parameters in ruminally cannulated non-lactating dairy cows that were adapted or unadapted to a high concentrate diet.

**Methods:**

A double 3×3 Latin square design was used with three PAP treatments (control, powdered, and liquid PAP) and two adaptation protocols (adapted, unadapted; applied to the square). Adapted animals were transitioned for 2 weeks from an all-forage to an 80% concentrate diet, while unadapted animals were switched abruptly.

**Results:**

Interactions between sampling time and adaptation were observed; 12 h after feeding, the adapted group had lower ruminal pH and greater total short chain fatty acid concentrations than the unadapted group, while the opposite was observed after 24 h. Acetate:propionate ratio, molar proportion of butyrate and ammonia nitrogen concentration were generally greater in adapted than unadapted cattle up to 36 h after feeding. Adaptation promoted 3.5 times the number of *Entodinium* protozoa but copy numbers of *Streptococcus bovis* and *Fibrobacter succinogens* genes in rumen fluid were not affected. However, neither liquid nor powdered forms of PAP altered rumen acidosis variables in adapted or unadapted animals.

**Conclusion:**

Adaptation of cattle to highly fermentable carbohydrate diets promoted a more stable ruminal environment, but PAP was not effective in this study in which no animal experienced acute or sub-acute rumen acidosis.

## INTRODUCTION

Housing growing beef cattle in feedlots allows cattle to gain weight in a short period of time due to diet formulation and use of high-quality feeds, which decreases age at slaughter. With this, more meat is produced with less land area compared with extensive grazing systems. While use of grain-based feedlot diets is economical, transition from high-forage to high-concentrate diets presents a challenge for the animal. Abrupt change in the fermentable carbohydrate content of diets leads to ruminal pH and acetate:propionate (C2:C3) ratio reduction and total short chain fatty acid (SCFA) concentration increase which may destabilizes rumen population and causes drastic modifications in the ruminal environment [1]. In beef cattle, subacute and acute acidosis are considered to occur when ruminal pH decreases to less than 5.6 and 5.2, respectively [2]. The most common bacteria detected in the rumen of cattle with sub-acute ruminal acidosis (SARA) are *Lactobacillus* spp. and *Streptococcus bovis* (*S. bovis*) [3], which produce lactic acid, contributing to lower ruminal pH, while *Fusobacterium necrophorum* (*F. necrophorum*) causes liver abscesses and invades the ruminal epithelium causing ruminitis [4,5]. To minimize the risk of acidosis, animals are usually adapted gradually from a forage-based diet to a high-concentrate diet.

Additionally, ionophore antibiotics are widely used in feedlot diets for acidosis control through modulating rate of intake, ruminal fermentation, and reduce the risk of acidosis [5]. However, widespread use of antibiotics in animal feeds is of increasing concern due to resistance. Hence new feed additives that prevent digestive disturbances with low safety risk for humans consuming the animal products are desirable. One such new compound is an avian-derived polyclonal antibody preparation (PAP) against *S. bovis* and F*. necrophorum*. The theory is that when PAP is administered to cattle receiving high concentrate diets the antibodies act against specific ruminal bacteria that are associated with nutritional disorders such as acidosis [6]. Because the antibodies do not directly modify the DNA or RNA of the target organism, microbial resistance does not typically occur, or if it does occur, it is possible to create new antibodies from the resistant microorganism [7].

Marino et al [8] fed ruminally cannulated dry cows different energy sources and observed that a liquid form of PAP (against *S. bovis*, *F. necrophorum*, *Clostridium aminophilum* [*C. aminophilum*], *Peptostreptococcus anaerobius*, and *C. sticklandii*) was as efficient as monensin in preventing a decline in ruminal pH at the peak of fermentation. However, Bastos et al [9] and Pacheco et al [10] tested other types of PAP in a powdered form and did not observe any effect of these products. It is not clear whether the discrepancy among these studies is due to the PAP itself, or the form of the product.

The aim of the present experiment was to evaluate the effects of liquid and powdered forms of an experimental PAP, with antibodies mainly against the ruminal bacteria *S. bovis* and *F. necrophorum*, on ruminal fermentation in ruminally cannulated cows adapted or abruptly changed to a highly fermentable concentrate (HFC) diet.

## MATERIALS AND METHODS

### Experimental design and treatments

The guidelines established by University of São Paulo (Brazil) Ethic Committee on Animal Use of the Research (CEEA) – n° 1982/2010 were followed when taking care of the cows. The study was conducted at the College of Veterinary Medicine and Animal Science, University of São Paulo, Brazil. Six Holstein nonpregnant and nonlactating dairy cows (body weight [BW]: 744±85 kg) (mean±standard deviation) previously fitted with ruminal cannulas were housed in individual stalls with sand bedding, a feed bunk, and access to drinking water within a well-ventilated facility. The cows were randomly assigned to two 3×3 Latin squares with a factorial arrangement of treatments; 3 PAP treatments allocated within square ×2 adaptation protocols applied by square (adapted, unadapted). The three treatments were: control (CON, no PAP), powdered form of PAP (PAPP), and liquid form of PAP (PAPL). The cattle in the adapted square received a series of diets (forage:concentrate [F:C]): 100:0 from d 0 to d 4; 70:30 from d 5 to d 9; 40:60 from d 10 to d 14; and 20:80 from d 15 to d 17. The cattle in the unadapted square received the 100:0 diet from d 0 to d 14 and then the final 20:80 diet from d 15 to d 17. Each of the 3 periods was 17 d long and sampling started immediately after feeding on d 15. The animals were weighed on the first and last day of each experimental period.

Diets were offered as total mixed rations twice a day at 0800 and 1600 h for *ad libitum* intake. All feed bunks were examined every morning at 0700 h. If there was no feed remaining in the feeder, the amount offered was raised by 10%. If up to 10% remained, the amount of feed offered was not changed and if the surplus was >10%, the feed offered was reduced by 10%. The forage source was fresh sugarcane chopped with a theoretical mean particle size of 1.24 cm. The composition of the experimental diets is presented in Table 1. The diet formulations were evaluated using the Cornell Net Carbohydrate and Protein System 6.5.

The PAP preparations were administered through the ruminal cannula twice a day, starting at d 1, just before meals. The PAPP was administered via absorbent tissue paper (7 g/d; 3.5 g each time) and PAPL was administered (21 mL/d; 10.5 mL each time) using a plastic syringe. The quantities of the 2 PAP presentations were equivalent on a dry matter (DM) basis. Procedures for generating the PAP evaluated in this study were similar to those described by DiLorenzo et al [6,11] and Marino et al [8]. Polyclonal antibodies were produced by CAMAS Inc. (Le Centre, MN, USA). The final product contained approximately 46.0% of antibodies against *S. bovis* (ATCC 9809), 23.0% against *F. necrophorum* (ATCC 27852), 15.4% against *Escherichia coli* (*E. coli*) O157:H7 and 15.4% against endotoxins. The same blend of microorganisms was used to generate the liquid and powdered forms of PAP, but the powdered form was obtained by spray-drying. Storage throughout the experiment was in a hermetically sealed package to protect it from light and heat. The liquid form of PAP was stored at 2°C to 8°C and it was also protected from light throughout the experiment.

### Sample collection and laboratory methods

Feed samples were dried at 55°C for 72 h and ground to pass through a 1-mm screen to determine DM (method 934.01 [12]); organic matter (OM, method 924.05 [12]); crude protein (CP) by total N determination using the micro-Kjeldahl technique (method 920.87 [12]); ether extract (EE) determined gravimetrically after extraction using petroleum ether in a Soxhlet extractor (method 920.85 [12]) and neutral detergent fiber (NDF, with heat-stable α-amylase) according to Van Soest et al [13]. The value of nonfiber carbohydrates (NFC) was estimated as: NFC (% DM) = 100−(CP+NDF+ EE+ash).

### Ruminal fermentation parameters

Ruminal fluid samples were collected through the ruminal cannula using a vacuum pump at 0, 3, 6, 9, 12, and 24 h after the morning meal on d 15 and d 16 of each period, except for 0 h sample, which was collected before the morning meal. The evening meal was provided after the 12 h collection. At the results section and figures, it is described as 0, 3, 6, 9, 12, 24, 27, 30, 33, and 36 h to be easy to interpret as 2 consecutive days. Approximately 500 mL of ruminal fluid was collected at each sampling time by sampling 3 different locations within the rumen. Immediately after the collection, 100 mL of ruminal fluid was used for pH determination with a portable digital pH meter (HANNA instruments Limited HI8424, Bedfordshire, UK). The SCFA analyses included acetate, propionate and butyrate, and were measured by gas chromatography according to Erwin et al [14]. Total lactic acid concentration was measured colorimetrically according to Pryce [15]. Ammonia-nitrogen (NH_3_-N) concentration was determined using the method described by Kulasek [16] as adapted by Foldager [17].

### Ruminal bacteria and protozoa

Samples of liquid and solid rumen contents were collected 3 h after the morning meal on d 16 of each period from three different locations in the rumen. Samples were manually mixed and separated into two 25-mL aliquots of each. Sample processing was performed as described by Stevenson and Weimer [18]. Thereafter, the bacteria pellet was dissolved in 700 μL buffer and kept at −80°C until analysis. The DNA was extracted from duplicate subsamples (100 μL) of each rumen sample using a Qiagen DNA stool mini kit (QIAGEN, Hilden, Germany). Real-time polymerase chain reaction (PCR) was carried out (7500 Real-Time PCR System, Applied Biosystems, Forster City, CA, USA) using 96-well plates and water as a negative control. In each reaction mixture, 1× concentration of SYBR Green (Applied Biosystems, USA), 300 nM of each primer, 6.6 μL of nuclease-free water and 1 μL of DNA template were used totalling 24 μL. The primer sequences are presented in Table 2. The real-time PCR amplification cycle included an initial denaturation step at 95°C for 10 min, followed by 44 cycles of heating and cooling at 95°C for 15 s and 60°C for 30 s, and extension at 72°C for 30 s. Melting curve analyses was used to evaluate the amplicon specificity. The relative quantification of target bacteria population to a reference sample was assessed using the 2^−ΔΔCt^ method [19]. Using the reaction efficiency analysis proposed by Yuan et al [20], it was determined that all primers functioned with efficiency not different from 100%.

For total and differential counts of ruminal protozoa 10 mL of ruminal contents was collected from the ventral sac 3 h after the morning meal on d 16 of each period and stored in glass vials with 20 mL of 18.5% formaldehyde. Subsequently, the sample was stained with two drops of 2% brilliant green and diluted; protozoa were identified (genus *Isotricha*, *Dasytricha*, *Entodinium*, and *Diplodiniinae* subfamily) and counted using a Neubauer Improved Bright-Line counting chamber (Hausser Scientific Partnership, Horsham, PA, USA) by optical microscopy (Olympus CH-2, Tokyo, Japan) [21].

### Blood acute phase proteins

Blood samples for haptoglobin (Hp) determination were taken from the caudal vein using a Vacutainer (BD-Becton, Dickinson and Company, Franklin Lakes, NJ, USA) containing sodium heparin as an anticoagulant. Samples were collected on d 17 of each period just before feeding. A 10 mL blood sample was collected and maintained on ice until centrifuged at 3,000×g at 4°C for 20 min to separate the plasma. Plasma samples were stored at −20°C until analysis. The Hp was determined using the ALPCO IMUMUNOASSAYS ELISA test (Catalog number: 41-HAPBO-E01; Salem, MA, USA). The plasma was diluted 1:10 (vol/vol) with phosphate buffered saline and centrifuged. An aliquot (100 μL) of diluted plasma was placed in duplicate in the microtitration plate. The enzyme-antibody conjugate was added (100 μL) to the sample in the well of the plate. In addition, 100 μL of the chromogenic substrate solution was added at room temperature. The absorbance was read after 10 min at 450 nm using a microplate reader (Thermo Scientific Multiskan FC Microplate Photometer, Vantaa, Uusimaa, Finland).

### Statistical analyses

Data were analyzed by Statistical Analysis System software (SAS Inst., Inc., Cary, NC, USA) using a mixed model that included the fixed effects of feed additive, adaptation, and the interaction between feed additive and adaptation. In this design, the effects of adaptation protocol are confounded with square. However, both squares were conducted simultaneously and animals in both squares were similar (age, initial weight and genetics) to minimize differences between squares. The effects of period and animal nested within adaptation were considered random factors. The variables ruminal pH, total concentration and molar proportion of SCFA, lactate and NH_3_-N concentrations were analyzed with repeated measures to account for time of sampling. In this case, the model accounted for the effects as described above plus time and their interactions with feed additive and adaptation. Effects were considered significant at p<0.05. For the analysis, 15 covariance structures were used, and the best fit structure was chosen based on it having the lowest Akaike information corrected criterion. Effects were separated by Tukey test.

## RESULTS

**Figure 1:**
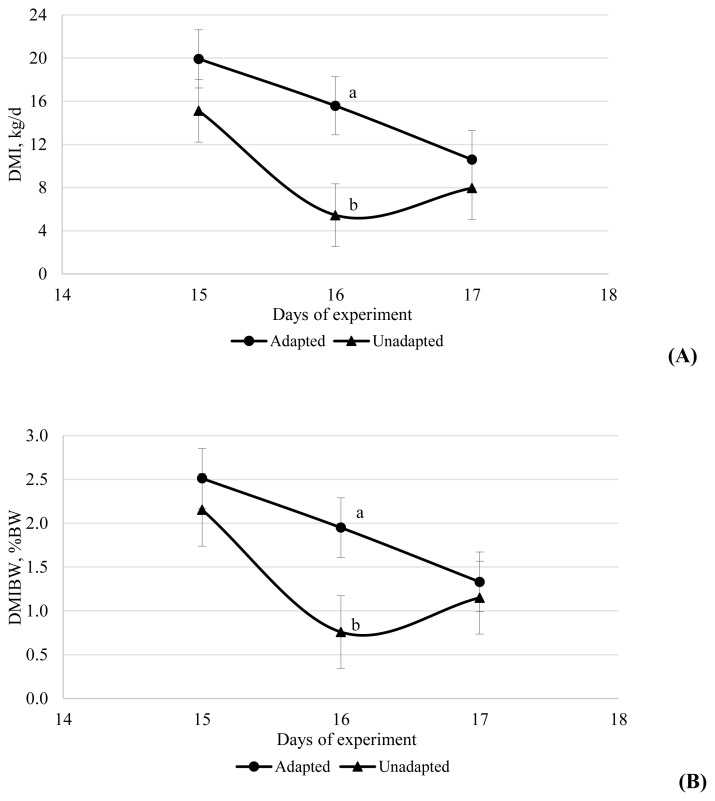
Dry matter intake (DMI, kg/d) (A) and DMI as a percentage of body weight (DMBW, % BW) (B) on day 15, 16, and 17 of the study for cattle adapted or not adapted to highly fermentable carbohydrate diets. The adapted cattle received a series of diets of increasing forage:concentrate ratio from day 1 to 14 with the final high concentrate diet fed from day 15 onwards, whereas the unadapted cattle received a forage diet from day 0 to 14 with the final high concentrate diet fed from day 15 onwards.

An interaction between time×adaptation was observed for dry matter intake (DMI) because the adapted group had greater DMI on 16 d compared with the unadapted group (15.58 vs 5.45 kg/d; 1.95 vs 0.76% of BW; Figure 1).

**Figure 2:**
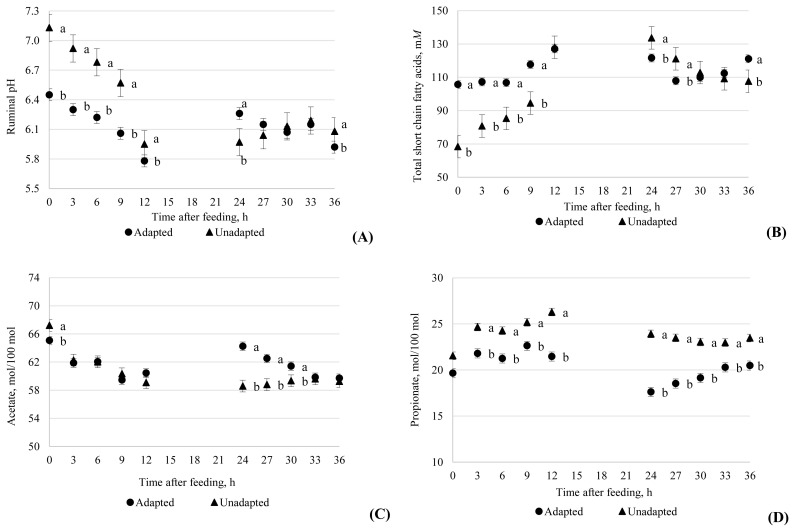
Ruminal pH (A), total short chain fatty acids (m*M*) (B), acetate (% molar proportion) (C) and propionate (% molar proportion) (D) responses to two adaptation protocols to highly fermentable carbohydrate diets. Time refers to hours after the morning meal on day 15. The adapted cattle received a series of diets of increasing forage:concentrate ratio from day 1 to 14 with the final high concentrate diet fed from day 15 onwards, whereas the unadapted cattle received a forage diet from day 0 to 14 with the final high concentrate diet fed from day 15 onwards.

There were no interactions between additive treatment and adaptation protocol for any of the ruminal fermentation variables. The main effects of treatment are presented in Table 3. For ruminal pH, a sampling time×adaptation interaction was observed (p<0.001; Table 3). Up to 36 h after feeding a diet with 80% concentrate, the unadapted group had greater pH values compared to the adapted group except for 24 h post feeding (Figure 2A). The PAP treatments had no effect on ruminal pH.

**Figure 3:**
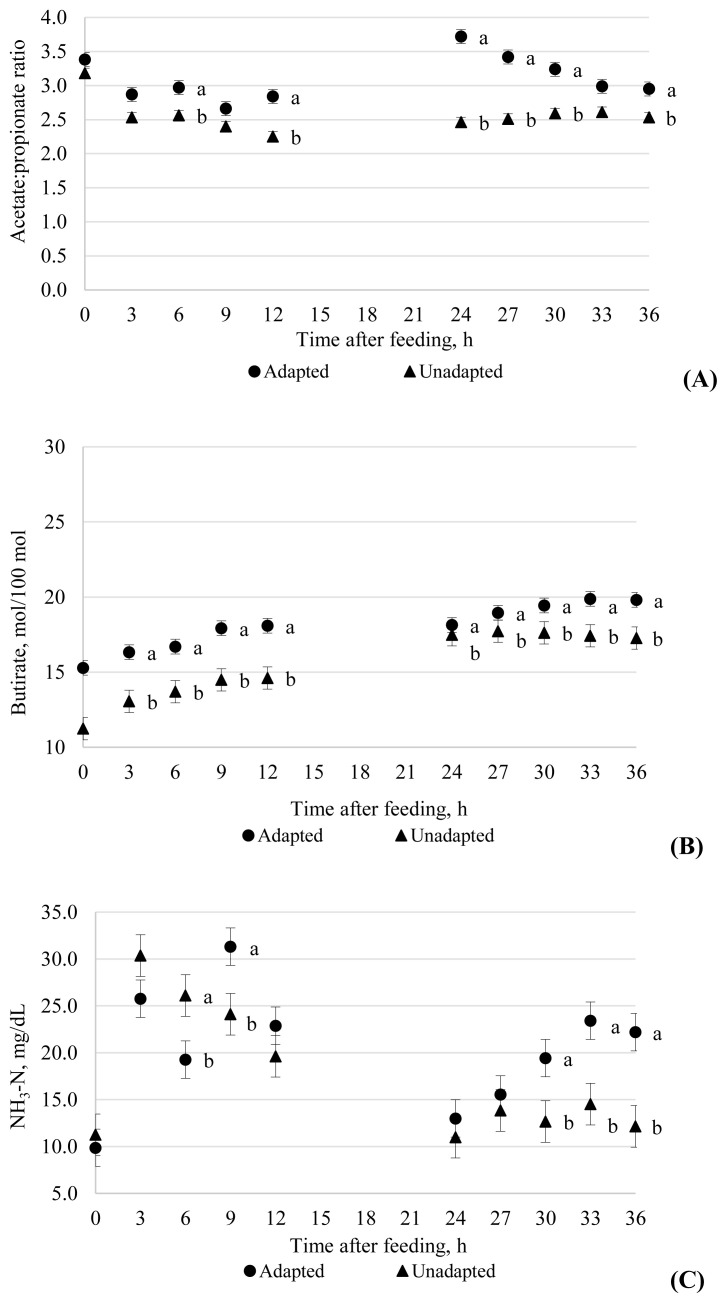
Acetate:propionate ratio (mol/mol) (A), butyrate (% molar proportion) (B) and NH_3_-N (mg/dL) (C) responses to two adaptation protocols to highly fermentable carbohydrate diets. Time refers to hours after the morning meal on day 15. The adapted cattle received a series of diets of increasing forage:concentrate ratio from day 1 to 14 with the final high concentrate diet fed from day 15 onwards, whereas the unadapted cattle received a forage diet from day 0 to 14 with the final high concentrate diet fed from day 15 onwards.

A sampling time×adaptation interaction was observed for total SCFA concentration (p<0.001; Table 3). At 0, 3, 6, 9, and 36 h post feeding, the adapted group had greater concentrations of SCFA compared to the unadapted group (Figure 2B). Only at 24 and 27 h post feeding of a diet with 80% concentrate was the inverse observed (121.6 vs 133.7 and 107.9 vs 121.1 m*M*). Similarly, there were sampling time×adaptation interactions (p<0.001) for acetate, propionate and butyrate molar proportions (Table 3). For acetate, the unadapted group had greater proportions than the adapted group 0 h post feeding (67.2 vs 65.1 mol/100 moles), but at 24, 27, and 30 h, the adapted group had greater proportions than the unadapted group (Figure 2C). For propionate, the unadapted group had greater proportions compared with the adapted group at 3, 6, 9, 12, 24, 27, 30, 33, and 36 h post feeding (Figure 2D). The acetate:propionate (C2:C3) ratio at 6, 12, and 36 h post feeding was greater for adapted than unadapted cattle (Figure 3A). The relative difference between adapted and unadapted cattle was much greater at 24, 27, and 30 h post feeding (3.72 vs 2.46, 3.42 vs 2.51, 3.24 vs 2.59, respectively). At 0, 3, 6, 9, 12, 33, and 36 h post feeding, adapted cows had greater butyrate proportions than unadapted cows (Figure 3B).

For lactate, there was no time×additive interaction, and no effect of additive or adaptation was observed (Table 3). However, for NH_3_-N concentration, a time×adaptation interaction (p<0.001) occurred (Figure 3C). At 6 h post feeding, the unadapted group had greater values than the adapted group (26.1 vs 19.3 mg/dL, respectively). At 9, 30, 33, and 36 h post feeding, the adapted group had greater values compared to the unadapted group (31.3 vs 24.2, 19.4 vs 12.7, 23.4 vs 14.5, and 22.2 vs 12.2 mg/dL, respectively). No effect of PAP treatment was observed for lactate or NH_3_-N concentration.

There was no effect of adaptation or PAP treatment on the expression of *S. bovis* and *Fibrobacter succinogens* populations, and there were no interactions between additive and adaptation (Table 4). Total counts of ruminal protozoa were greater (p = 0.005) in adapted than unadapted cattle. No effect of PAP treatment was observed. There were no treatment effects for Hp.

## DISCUSSION

The greater DMI of adapted cattle following the introduction of the HFC diet on day 15 would suggest a benefit of adaptation, possibly due to a reduction in digestive disturbances. However, the lack of difference between treatments on day 17 indicates the benefit was short-term. The short-term decrease in DMI following the introduction of a HFC diet corroborates the findings of Holtshausen et al [22], where DMI of feedlot cattle decreased the day following introduction of a 90% concentrate diet with no difference in intake thereafter. The lack of effect of PAP on DMI in the current study corroborates the lack of difference in DMI reported by DiLorenzo et al [11]. The relatively minor effects on DMI of the adaptation protocol and the lack of effect of PAP on DMI in the present study may indicate that the conditions used did not lead to a substantial occurrence of digestive disturbance, which is confirmed by the rumen fermentation variables presented.

Supplying HFC diets to cattle receiving forage diets stimulates the growth of amylolytic ruminal bacteria, increases SCFA production and decreases ruminal pH. These changes typically occur within 12 to 24 h of the animal receiving the diet [2]. In the present experiment, unadapted cattle had greater ruminal pH than adapted cattle during the first 12 h post feeding HFC, indicating there was a delay before abrupt feeding of HFC caused a decline in ruminal pH. The greater ruminal pH of the unadapted cattle corresponded to the short-term decrease in DMI, and subsequent decrease in SCFA, following the introduction of the HFC diet. The lower pH at 24 h for the unadapted group compared with the adapted group suggests that abrupt feeding of HFC without adaptation destabilized the rumen, indicating that the resulting pH was a function of the diet as well as the adaptation protocol. However, the similar pH of the two groups at subsequent samplings indicates the destabilization of pH and DMI was temporary.

The lack of effect of PAP, regardless of whether it was in a liquid or powdered form, may indicate that the treatment duration used in the study was not long enough. It is, however, more likely that the lack of effect was due to the lack of occurrence of acute or subacute acidosis. The lowest ruminal pH value observed during the study was 5.58, which occurred in an animal from the adapted group 12 h post feeding. According to Nagaraja and Titgemeyer [5], an animal is considered to have acute acidosis when ruminal pH reaches 5.0 or less, and subacute acidosis when ruminal pH is between 5.0 and 5.5. Owens [2] suggested a threshold value of 5.2 and 5.6, respectively, for acute and subacute acidosis. Based on these criteria, no animal experienced subacute or acute acidosis in the study, which might explain the lack of PAP treatment effects on DMI and variables used to assess acidosis. The lack of response to liquid and powdered forms of PAP may also suggest that the feed additive was ineffective, regardless of physical form. The results in the literature on the effects of feeding PAP to cattle on ruminal pH are contradictory. When comparing PAP (against *S. bovis*, *F. necrophorum*, *C. aminophilum*, *Peptostreptococcus anaerobius*, and *C. sticklandii*) to monensin, Marino et al [8] observed an interaction between PAP treatment and time. In that study the pH was similar for PAP and control groups, with lower pH for the monensin group, while at 2 and 4 h post feeding, both treatment groups had greater pH values than the control group. Working with animals adapted to high concentrate diets, DiLorenzo et al [6] did not observe any effect of feeding PAP on ruminal pH. Blanch et al [23] observed greater pH values at 6, 8, and 9 days after a high concentrate feeding challenge in animals fed PAP compared to those with no additive.

Total SCFA concentrations observed in the study were consistent with expected levels of 100 to 160 mM in the rumen of cattle receiving HFC diets (70% to 90% concentrates [24,1]). The results indicate that total SCFA concentration was relatively stable for the adapted group, whereas the concentrations increased substantially for the first 12 h in the unadapted group after they were presented with HFC diet. The lack of effect of PAP on total SCFA concentration is consistent with the lack of effects on DMI and rumen pH. In contrast, Blanch et al [23] reported that PAP fed animals had greater values of total SCFA than the control group. Goad et al [24] compared animals adapted to a high-forage diet (20% of concentrate) to those adapted to a high-concentrate (80%) diet when both groups were given a challenge diet that consisted of 100% concentrate and the authors observed that the molar proportion of acetate decreased in both groups. These findings corroborate the results of the present study whereby molar proportion of acetate for the unadapted group decreased from 67.2 mol/100 mol at 0 h to 59.3 mol/100 mol at 36 h whereas for the adapted group it decreased from 65.1 mol/100 at 0 h to 59.7 mol/100 mol at 36 h.

In a diet adaptation study, Bevans et al [1] observed that propionate molar proportion increased when cattle received one intermediary diet of 65% concentrate compared with cattle that were gradually adapted using five intermediary diets (48.3%, 56.7%, 65.0%, 73.3%, and 81.7% concentrate). These findings corroborate the current experiment where the adapted group had lower proportion of propionate. Goad et al [24] observed a greater decline in C2:C3 in cattle fed 20% concentrate when abruptly fed 100% concentrate, compared with cattle fed 80% concentrate before being changed to 100% concentrate. The C2:C3 of animals fed 20% concentrate reduced from 5.5:1 at 0 h to 1.4:1 at 72 h after receiving 100% concentrate, whereas for cattle adapted to the 80% concentrate diet, C2:C3 ratio reduced from 3.8:1 at 0 h to 0.9:1 at 72 h after receiving the 100% concentrate diet. The value of 0.9 in the latter group occurred when ruminal pH was below 5.5, an indication that the metabolic routes for propionate production are stimulated at lower pH. In the present study, despite a decrease in C2:C3 ratio after the diet was switched (12 h for unadapted group and 9 h for adapted group), the ratio did not reach the low values observed in the study of Goad et al [24], indicating that the challenge in our study was considerably less severe.

Lactate producing bacteria such as *S. bovis* and *Lactobacillus* spp. proliferate below pH of 5.5 [25,26]. Simultaneously, growth of the lactate utilizing bacteria *Megasphera elsdenii* and *Selenomonas ruminantium* is inhibited [27]. Independent from the type of diet, a ruminal pH below 5.5 is expected to increase lactate concentration. In the current experiment, as ruminal pH did not reach values below 5.5, no PAP effect was expected on this variable. A similar lack of effect of feeding PAP to cattle on lactate concentration was reported by DiLorenzo et al [6] and Blanch et al [23]. The lack of effect of PAP additive on NH_3_-N concentration corroborates the findings of other studies [23,9].

As the proportion of concentrate in a diet increases, a decrease in cellulolytic bacteria, such as *F. succinogens*, and an increase in acid tolerant bacteria such as *S. bovis* [28,29] is expected. Fernando et al [30] observed an increase in the population of *S. bovis* at the beginning of adaptation, but at the end of adaptation this difference was not significant, arguing that using a step-up diet to adapt cattle to a high concentrate diet would control the population of *S. bovis*. The population of *S. bovis* in animals adapted to concentrate diets has been reported to be similar to populations in cattle fed a forage diet [5]. Lack of effect of adaptation and PAP treatment on bacterial populations is consistent with the relatively high ruminal pH values observed (>5.5).

The larger *Entodinium* population in the ruminal fluid of the adapted cattle indicates that the ruminal protozoa may have been better adapted to the HFC diet. Arcuri et al [29] reported increased *Entodinium* population in animals fed high-starch diets while Carvalho et al [31] reported the *Entodinium* population was positively correlated to total digestible nutrient content and negatively to NDF content of the diet. According to Otero [32], PAP acts by increasing the population of *Isotricha* because it decreases the numbers of acidifying bacteria of the rumen making the rumen favorable to the growth of *Isotricha*. However, that did not occur in the present study possibly because ruminal pH was not low enough to affect the population of this protozoa.

Haptoglobin values were within the normal range for cattle (<50 μg/mL [32]), and the lack of treatment effect indicated no inflammation associated with acidosis. Acute phase proteins such as Hp can be indicators of inflammation caused in animals [33] with concentrations greater than 200 μg/mL indicative of mild infection in cattle [34]. Comparing PAP (against *S. bovis*, *F. necrophorum*, *E. coli*, and several strains of proteolytic bacteria) with monensin, Pacheco et al [10] reported a lower incidence of rumenitis in animals receiving PAP. In the current experiment, ruminal pH remained relatively high and Hp values (<2 μg/mL) were low, thus there was no indication of inflammation from acidosis. It appears that the lack of PAP treatment effect on Hp is consistent with the general lack of acidosis in the animals regardless of adaptation protocol.

Overall, the degree of rumen acidosis that occurred in this study following introduction of the HFC diet was relatively mild and may not have provided ideal conditions for evaluating PAP. However, the conditions used were representative of those used by many commercial feedlots. It may be more effective in future studies to use an acidosis challenge model to evaluate PAP, wherein acute acidosis is induced by withholding feed for a short period of time (e.g., 12 h), followed by overfeeding of concentrate [38]. Nevertheless, the present study indicates that under conditions of mild rumen acidosis there is no short-term benefit from providing PAP to cattle to assist with transition from a high forage to high concentrate diet.

## CONCLUSION

Under the conditions of this study, adaptation of cattle to a high concentrate diet helped maintain high DMI when cattle were switched to HFC, which resulted in increased total SCFA, decreased rumen pH, increased C2:C3 ratio, and increased total protozoa counts for adapted compared with unadapted cattle. Adaptation of cattle to HFC resulted in short-term benefits in terms of DMI and rumen fermentation, but neither group experienced SARA during this study. Provision of PAP, either in powdered or liquid form, had no effect on any of the variables measured. Lack of effect of the polyclonal antibodies may have been due to the general lack of acute and subacute acidosis experienced by the cattle in the study, even when animals were not adapted to the high grain diet.

## Figures and Tables

**Table 1:** Composition and analysed nutrient content of experimental diets

Item	Diets (% of concentrate)			

	0	30	60	80
Ingredients (% of DM)				
Sugarcane, fresh and chopped	93.4	70.0	40.0	20.0
Dry-ground corn grain	-	12.5	46.9	70.2
Soybean meal	-	13.5	9.1	5.8
Urea	4.1	1.5	1.5	1.5
Salt	0.5	0.5	0.5	0.5
Vitamin and mineral premix1)	2.0	2.0	2.0	2.0
Nutrient content				
DM (%)2)	38.8	51.1	67.1	77.8
CP (% of DM)2)	14.3	15.1	14.5	15.2
Rumen degradable protein (% CP)3)	95.0	76.4	75.4	76.4
Rumen undegradable protein (%CP)3)	5.0	23.8	24.6	23.6
NDF (% of DM)2)	44.6	36.8	26.5	18.8
NDF of forage (% NDF)3)	54.1	40.7	23.3	11.6
Non-fiber carbohydrates (% of DM)2)	43.2	42.0	53.6	60.8
Total digestible nutrients (% of DM)3)	54.8	62.7	71.6	77.2
Ca (% of DM)3)	0.84	0.78	0.63	0.53
P (% of DM)3)	0.40	0.47	0.43	0.45

DM, dry matter; CP, crude protein; NDF, neutral detergent fiber.

1) Composition of vitamin and mineral premix per kilogram of product: 180 g of Ca, 130 g of P, 100 mg of Co, 1.250 mg of Cu, 2.200 mg of Fe, 90 mg of I, 2.000 mg of Mn, 15 mg of Se, 5.270 mg of Zn, and 1.300 mg of F (maximum).

2) Data of nutrient content were obtained by laboratory analysis.

3) Value estimated by Cornell Net Carbohydrate and Protein System 6.5.

**Table 2:** Specific primers used for the quantification of bacteria by polymerase chain reaction real time

Microorganism	16sRNA primers	Reference
Total bacteria	F: GTGSTGCAYGGYTGTCGTCA	[36]
	R:ACGTCRTCCMCACCTTCTC	
*Fibrobacter succinogenes*	F: GGTATGGGATGAGCTTGC	[37]
	R: GCCTGCCCCTGAACTATC	
*Streptococcus bovis*	F:TTCCTAGAGATAGGAAGTTTCTTCGG	[18]
	R:ATGATGGCAACTAACAATAGGGGT	

**Table 3:** Values of ruminal fermentation parameters for dry cows fed different preparations of PAP adapted or abruptly changed to highly fermentable carbohydrate diets

Variables	Adaptation (Adap)		Additive (Add)			SEM	p-value			
		
	Yes	No	CON	PAPL	PAPP		Adap	Add	Adap×Add	Time×Adap
pH	6.14	6.38	6.22	6.30	6.26	0.03	^**^	NS	NS	^***^
Concentration of SCFA (m*M*)										
Acetate (C2)	70.0	62.6	65.9	66.1	67.0	0.79	^*^	NS	NS	^***^
Propionate (C3)	23.1	24.9	23.9	23.9	24.2	0.39	NS	NS	NS	^***^
Butyrate	20.7	16.6	19.9	18.0	18.1	0.46	^**^	NS	NS	^***^
C2:C3 ratio	3.10	2.56	2.80	2.84	2.86	0.04	^*^	NS	NS	^***^
Total SCFA	113.8	104.1	109.6	108.0	109.2	1.43	^**^	NS	NS	^***^
Molar proportion of SCFA (mol/100 moles)										
Acetate	61.6	60.6	60.5	61.4	61.6	0.26	NS	NS	NS	^***^
Propionate	20.3	23.9	21.9	22.2	22.1	0.22	^**^	NS	NS	^***^
Butyrate	18.1	15.5	17.6	16.4	16.3	0.26	^*^	NS	NS	^**^
NH_3_-N (mg/dL)	20.3	17.6	19.6	19.0	18.2	0.66	^**^	NS	NS	^**^
Lactate (Mm/L)	0.31	0.33	0.28	0.33	0.32	0.03	NS	NS	NS	^**^

PAP, polyclonal antibody preparation; PAPL, liquid form of PAP; PAPP, powdered form of PAP; SEM, standard error of the mean; SCFA, short chain fatty acid.

Adap, adaptation protocol effect; Add, feed additive effect; Adap×Add, interaction between adaptation protocol and feed additive effect; Time×Adap, time of sampling and adaptation protocol effect.

NS, not significant; *, **, and *** for p<0.05, p<0.01, and p<0.001, respectively.

**Table 4:** Rumen bacteria and protozoa population and haptoglobin of dry cows fed different preparations of PAP adapted or abruptly changed to highly fermentable carbohydrate diets

Variables	Adaptation (Adap)		Additive (Add)			SEM	p-value		
		
	Yes	No	CON	PAPL	PAPP		Adap	Add	Adap×Add
Relative population1)									
*Fibrobacter succinogenes*	1.11	0.9	0.77	0.69	2.13	0.20	NS	NS	NS
*Streptococccus bovis*	0.87	1.16	1.13	0.71	1.27	0.22	NS	NS	NS
Total protozoa (×10^3^ mL^−1^)									
*Dasytricha*	12.9	17.4	13.7	14.3	17.5	2.26	NS	NS	NS
*Isotricha*	2.22	0.78	0.83	0.50	3.17	0.52	NS	NS	NS
*Diplodiniinae*	0.78	0.00	1.00	0.17	0	0.33	NS	NS	NS
*Entodinium*	197.9	56.1	139.0	78.2	163.8	35.2	*	NS	NS
Total/mL	214.2	74.2	155.2	93.3	184.2	36.5	*	NS	NS
Haptoglobin (μg/mL)	1.37	1.78	1.87	1.25	1.70	0.38	NS	NS	NS

PAP, polyclonal antibody preparation; PAPL, liquid form of PAP; PAPP, powdered form of PAP; SEM, standard error of the mean.

1) Changes in ruminal population based on the population size in control group.

NS, not significant;

* p<0.05.
